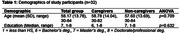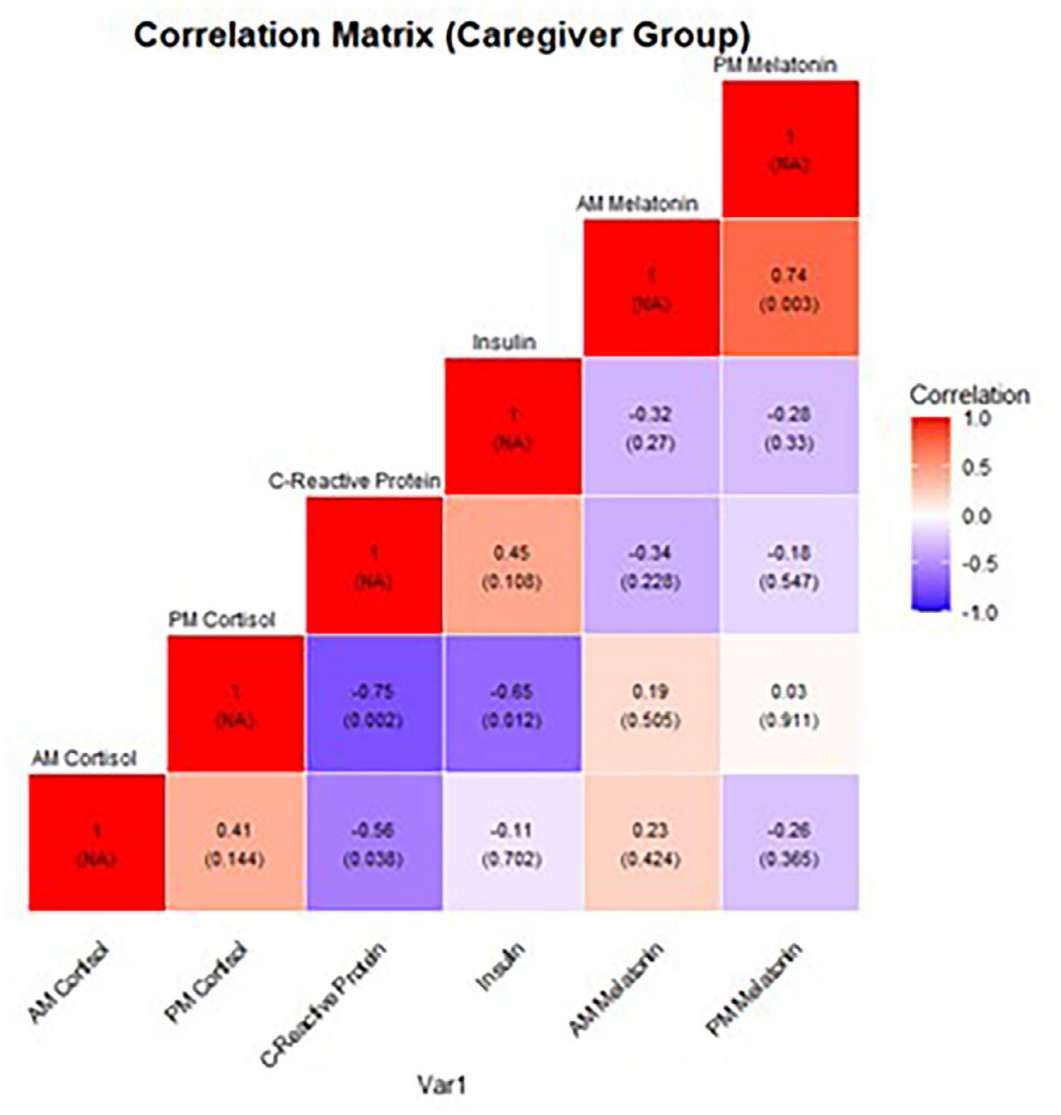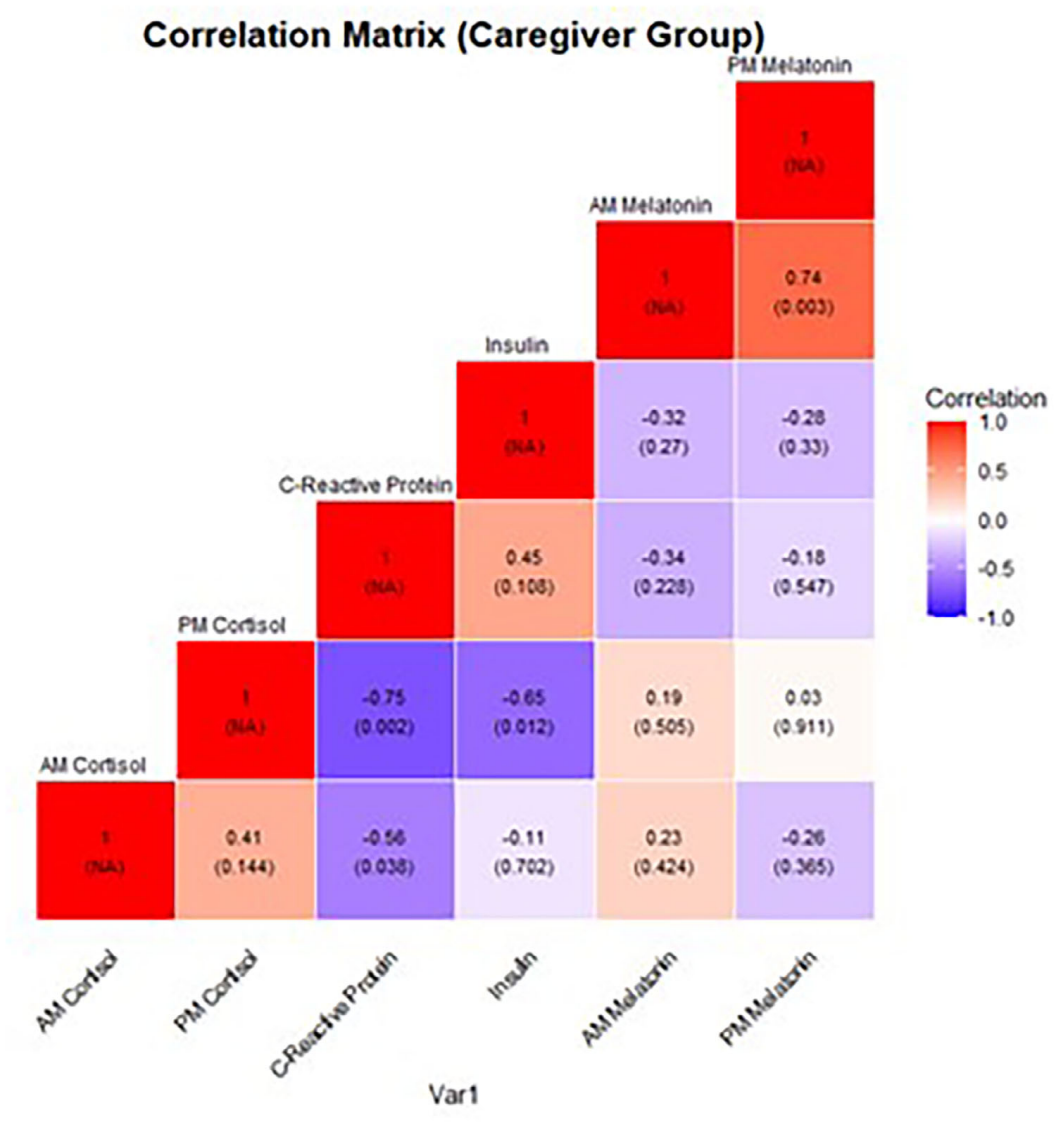# Performance of Salivary Biomarkers in Black Male Caregivers: An Exploratory Analysis

**DOI:** 10.1002/alz70856_105859

**Published:** 2026-01-07

**Authors:** Lilcelia A. Williams, Alexander Delong, Aaron P. Henry, Amani L Ali, Ann D Cohen, Robert W. Turner

**Affiliations:** ^1^ Department of Occupational Therapy, University of Pittsburgh, School of Health and Rehabilitation Sciences, Bridgeside Point I 100 Technology Drive, PA, USA; ^2^ Department of Medicine Division of Geriatric Medicine, University of Pittsburgh, School of Medicine, Pittsburgh, PA, USA; ^3^ University of Pittsburgh Alzheimer's Disease Research Center (ADRC), Pittsburgh, PA, USA; ^4^ George Washington University School of Medicine and Health Sciences, Washington, DC, USA; ^5^ The George Washington University, Washington, DC, USA; ^6^ The George Washington Univeristy School of Medicine and Health Sciences, Washington, DC, USA; ^7^ University of Pittsburgh Alzheimer's Disease Research Center, Pittsburgh, PA, USA; ^8^ University of Pittsburgh School of Medicine, Pittsburgh, PA, USA; ^9^ University of Pittsburgh, Pittsburgh, PA, USA; ^10^ Department of Psychiatry, University of Pittsburgh, Pittsburgh, PA, USA; ^11^ Duke University School of Medicine, Durham, NC, USA; ^12^ The George Washington University School of Medicine and Health Sciences, Washington, DC, USA

## Abstract

**Background:**

Non‐Hispanic Black American (NHBA) adults are twice as likely to be diagnosed with Alzheimer's disease (AD) at later stages and with greater severity than their White peers. Several reasons may contribute to these disparities, including worse overall health compared to peers in all other racial and ethnic groups in the United States. As a result, the burden of giving care for a loved one with Alzheimer's Disease or related dementias (AD/ADRD) may disproportionately impact NHBA individuals. While many research studies have cited the effect of providing care to a loved one with AD/ADRD, few studies have sought to understand the influence of caregiving on NHBA males. This cross‐sectional study aims to gain insight into how the burden of caregiving may uniquely impact NHBA male health conditions.

**Method:**

Caregivers and non‐caregivers who self‐identified as NHBA and male were enrolled between 2020 and 2024 (Table 1). Participants used a saliva collection kit to provide four samples for two consecutive mornings, at both AM and PM times, which were sent to Salimetrics, LLC for analysis. Samples with invalid results were removed for this preliminary analysis, reducing the sample size from 77 to 32. Samples were averaged across days and log transformed. The relationships between AM and PM Cortisol, AM and PM melatonin, C‐reactive protein (CRP), and Insulin were assessed using Pearson correlations (Figures 1‐2).

**Result:**

In the full sample set (*n* = 32), there was a significant correlation between (CRP) and PM cortisol (‐0.514, *p* = 0.0026) and between AM and PM melatonin (0.421, *p* = 0.0164). Amongst only caregivers (*n* = 14), there was a significant correlation between CRP and PM cortisol (‐0.746, *p* = 0.0022), CRP and AM cortisol (‐0.559, *p* = 0.0377), and between AM and PM melatonin (0.736, *p* = 0.0027). Insulin and PM cortisol (0.652, *p* = 0.0034) were correlated amongst non‐caregivers (*n* = 18).

**Conclusion:**

While this data is preliminary, dysregulated or lower cortisol due to stress has been shown to elevate average C‐reactive protein levels. Thus, the impact of stress from caregiving could increase chronic inflammation in such a way that it is not present in non‐caregivers. Our findings align with prior studies, reiterating the importance of identifying conditions that adversely impact NHBA male caregivers.